# Supplementary material for: DPPH Radical Scavenging Activity of New Phenolics from the Fermentation Broth of Mushroom Morehella importuna
Source: Molecules. 2023 Jun 14;28(12):4760. doi: 10.3390/molecules28124760 (PMC10303913; doi:10.3390/molecules28124760)
Supplement: Supplementary file 1 [file molecules-28-04760-s001.zip › molecules-2408951-supplementary.pdf]

Electronic Supplementary Information

DPPH radical scavenging activity of new phenolics from the fermentation broth of mushroom *Morehella importuna*

Feifei Wang [1,2], Jie Tan [1,2], Ruixiang Jiang [1,2], Feifei Li [1,2], Renqing Zheng [1,2], Linjun Yu [1,2], Lianzhong Luo [3,*], Yongbiao Zheng [1,2,*]

[1] Engineering Research Centre of Industrial Microbiology, Ministry of Education, College of Life Sciences, Fujian Normal University, Fuzhou 350117, China; yongbiaozheng@fjnu.edu.cn
[2] Provincial University Key Laboratory of Cellular Stress Response and Metabolic Regulation, College of Life Sciences, Fujian Normal University, Fuzhou 350117, China; yongbiaozheng@fjnu.edu.cn
[3] Engineering Research Center of Marine Biopharmaceutical Resource，Fujian Province University, Xiamen Medical College, Xiamen 361023, China; lianzhongluo@foxmail.com
* Correspondence: yongbiaozheng@fjnu.edu.cn (Y.B.Z); lianzhongluo@foxmail.com (L.Z.L.)

The List of Contents

No.	Content	Page
1	Figure S1 ^{1}H NMR spectra of compound **1**	2
2	Figure S2 ^{13}C NMR spectra of compound **1**	2
3	Figure S3 ^{1}H NMR spectra of compound **2**	3
4	Figure S4 ^{13}C NMR spectra of compound **2**	3
5	Figure S3 ^{1}H NMR spectra of compound **3**	4
6	Figure S4 ^{13}C NMR spectra of compound **3**	4
7	Figure S7 ^{1}H NMR spectra of compound **4**	5
8	Figure S8 ^{13}C NMR spectra of compound **4**	5
9	Figure S9 ^{1}H NMR spectra of compound **5**	6
10	Figure S10 ^{13}C NMR spectra of compound **5**	6
11	Figure S11 ^{1}H NMR spectra of compound **6**	7
12	Figure S12 ^{13}C NMR spectra of compound **6**	7
13	Figure S13 ^{1}H NMR spectra of compound **7**	8
14	Figure S14 ^{13}C NMR spectra of compound **7**	8
15	Figure S15 ^{1}H NMR spectra of compound **8**	9
16	Figure S16 ^{13}C NMR spectra of compound **8**	9
17	Figure S17 ^{1}H NMR spectra of compound **9**	10
18	Figure S18 ^{13}C NMR spectra of compound **9**	10
19	Figure S19 ^{1}H NMR spectra of compound **10**	11
20	Figure S20 ^{13}C NMR spectra of compound **10**	11
21	Figure S21 ITS DNA sequence of the strain *M. importuna*	12

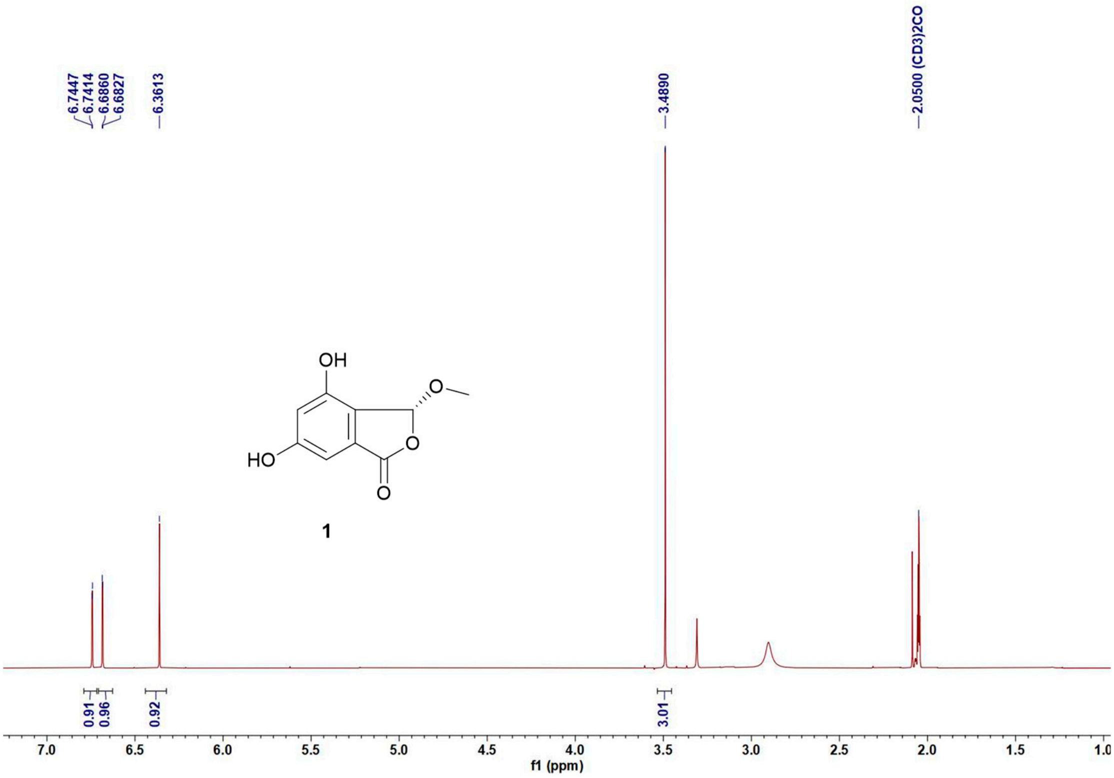

Figure S1 ^{1}H NMR spectra of compound **1** [Recorded at 600 MHz in $(CD_3)_2OD$]

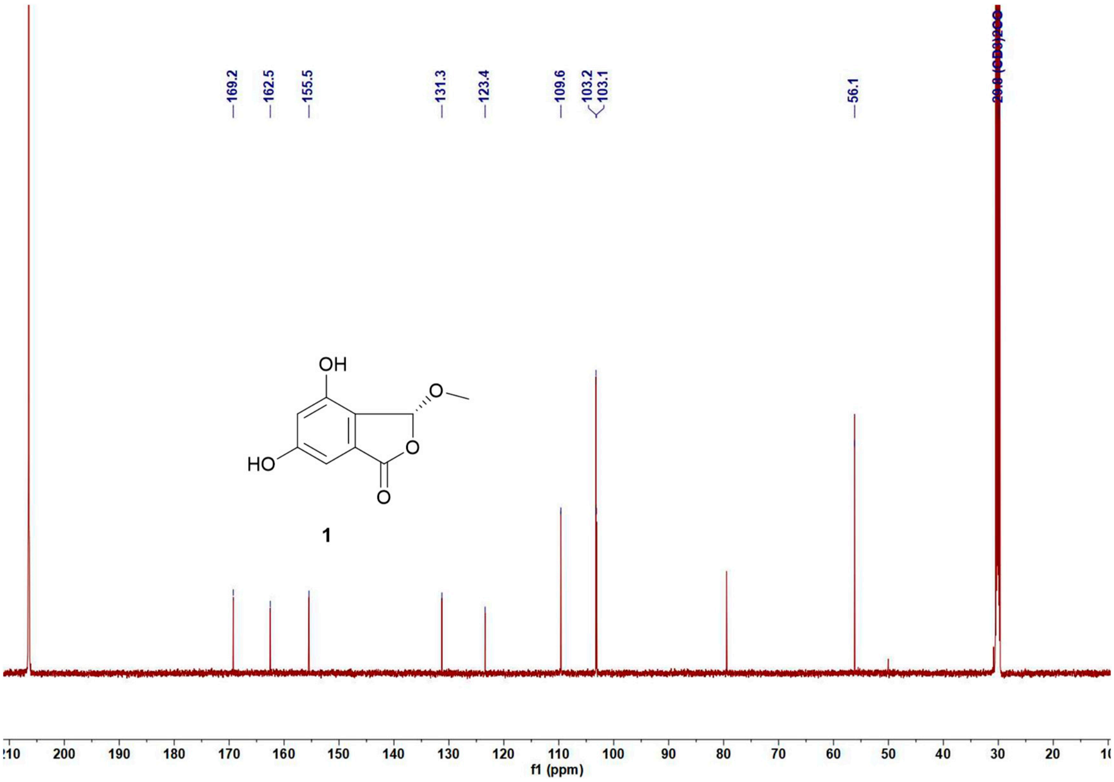

Figure S2 ^{13}C NMR spectra of compound **1** [Recorded at 150 MHz in $(CD_3)_2OD$]

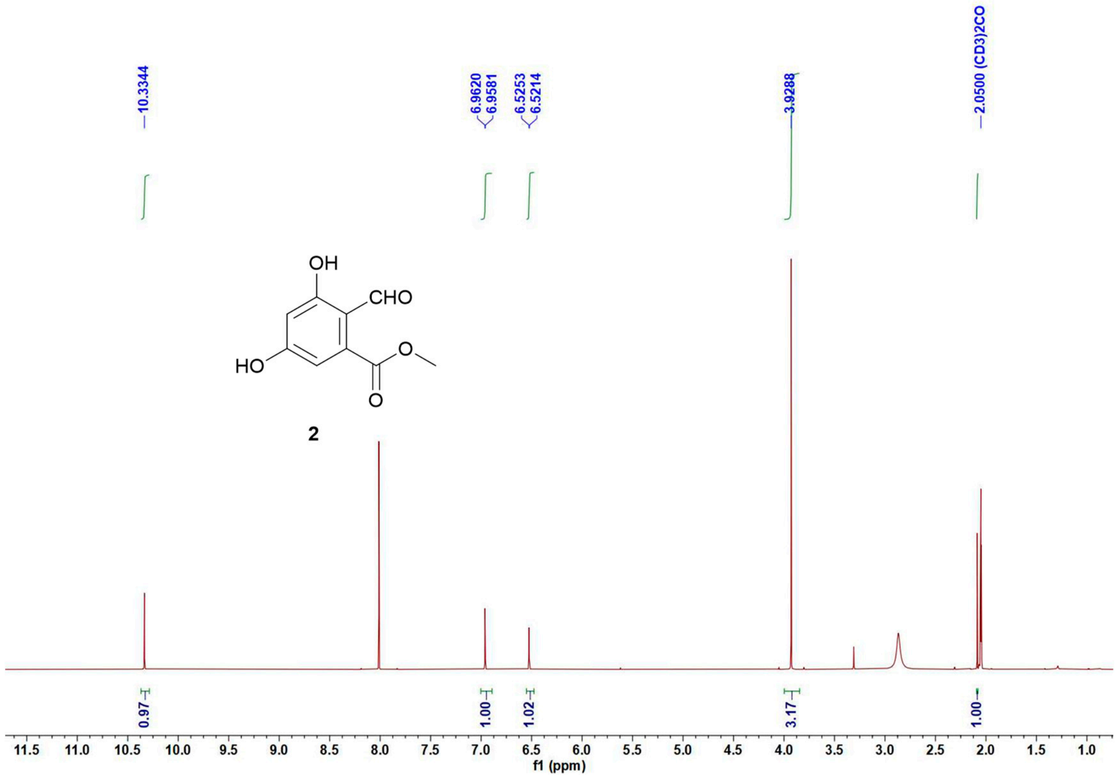

Figure S3 ^{1}H NMR spectra of compound **2** [Recorded at 600 MHz in $(CD_3)_2OD$]

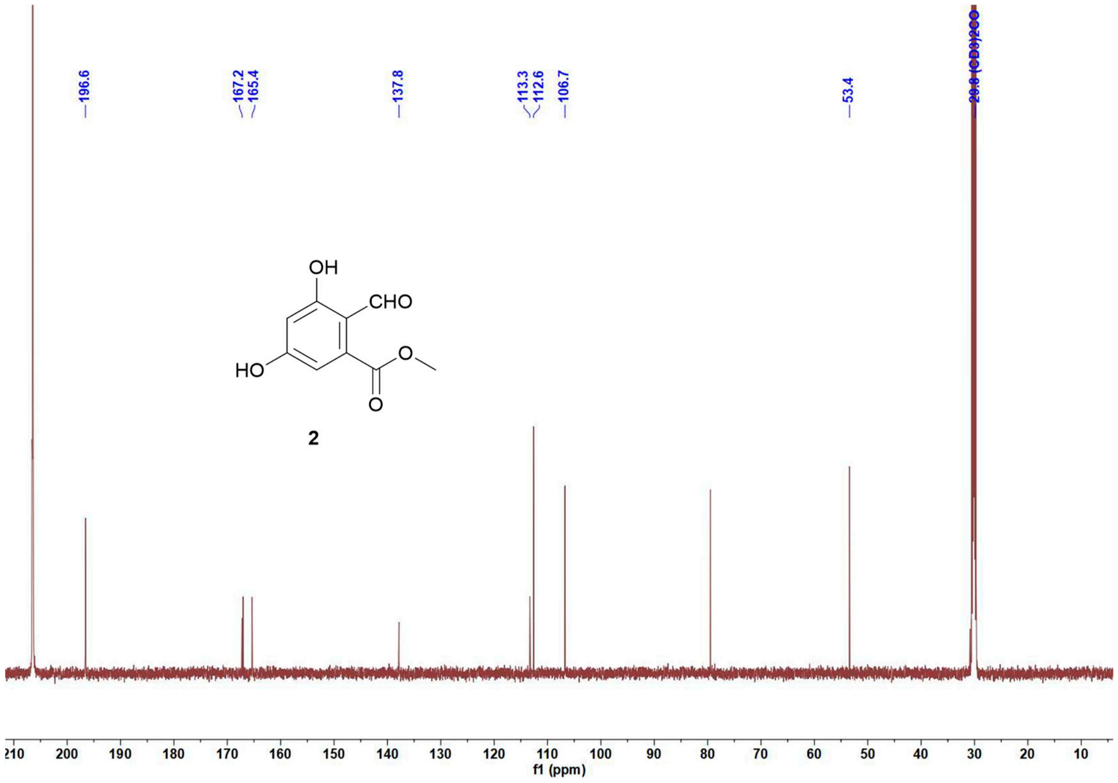

Figure S4 ^{13}C NMR spectra of compound **2** [Recorded at 150 MHz in $(CD_3)_2OD$]

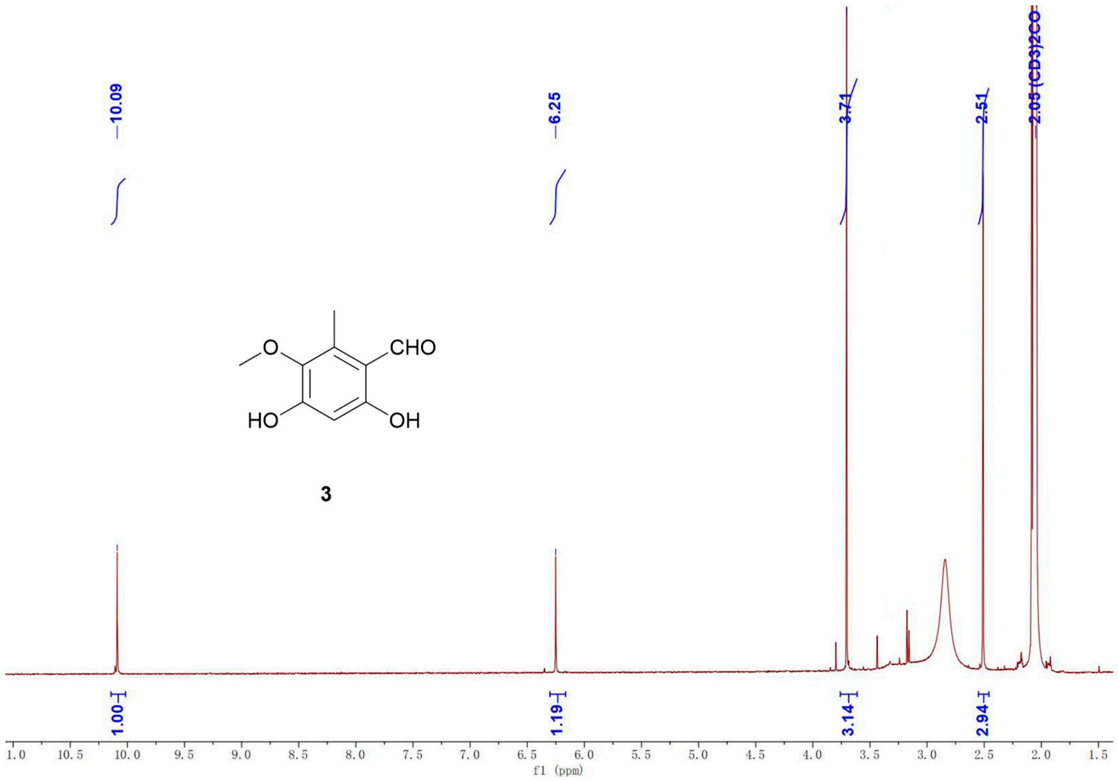

Figure S5 ^{1}H NMR spectra of compound **3** [Recorded at 500 MHz in $(CD_3)_2OD$]

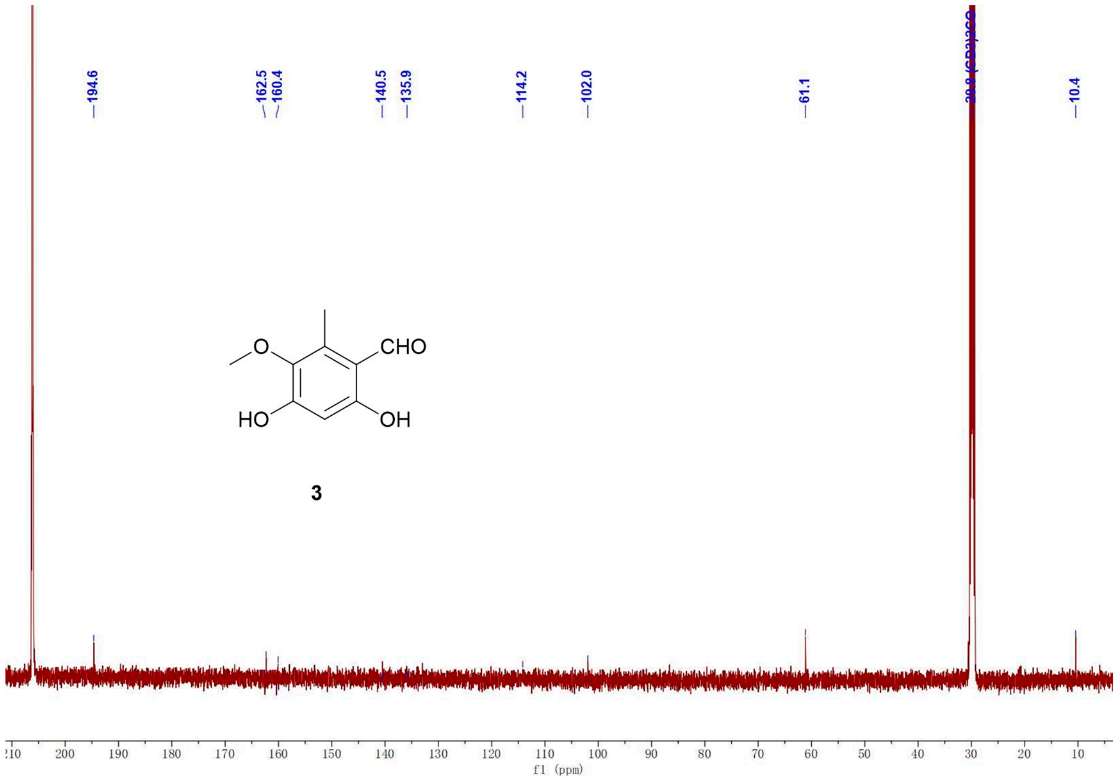

Figure S6 ^{13}C NMR spectra of compound **3** [Recorded at 125 MHz in $(CD_3)_2OD$]

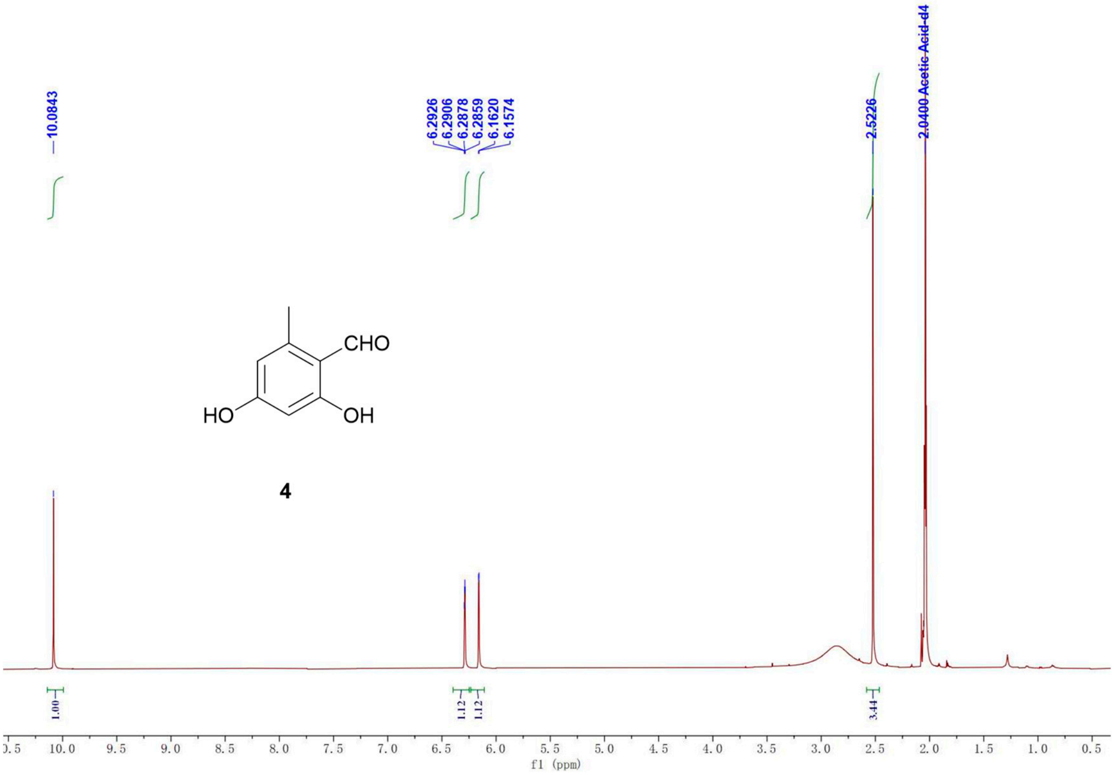

Figure S7 ^{1}H NMR spectra of compound **4** [Recorded at 500 MHz in $(CD_3)_2OD$]

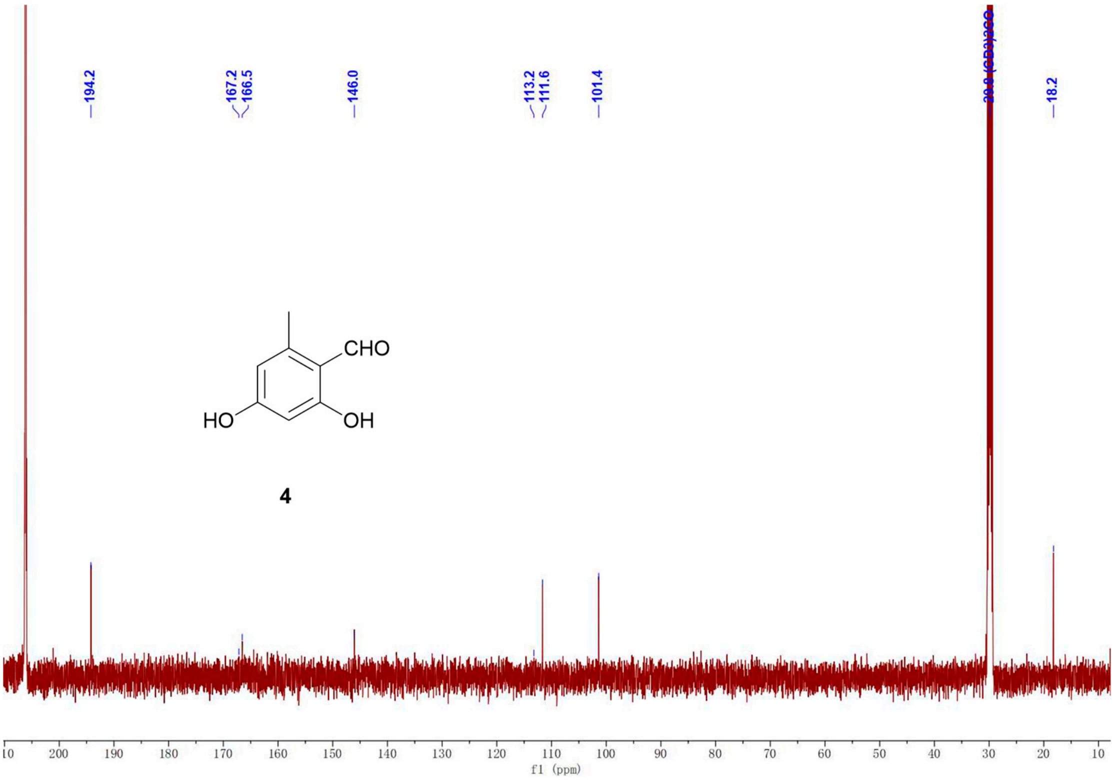

Figure S8 ^{13}C NMR spectra of compound **4** [Recorded at 125 MHz in $(CD_3)_2OD$]

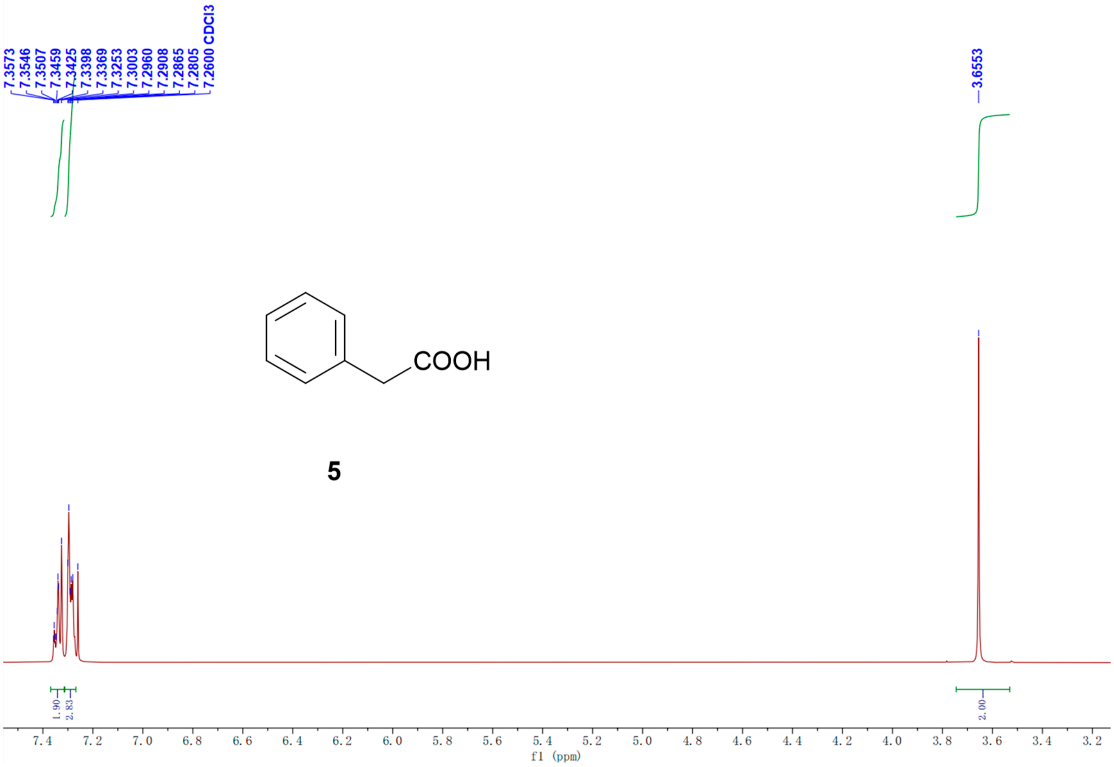

Figure S9 ^{1}H NMR spectra of compound **5** (Recorded at 500 MHz in $CDCl_3$)

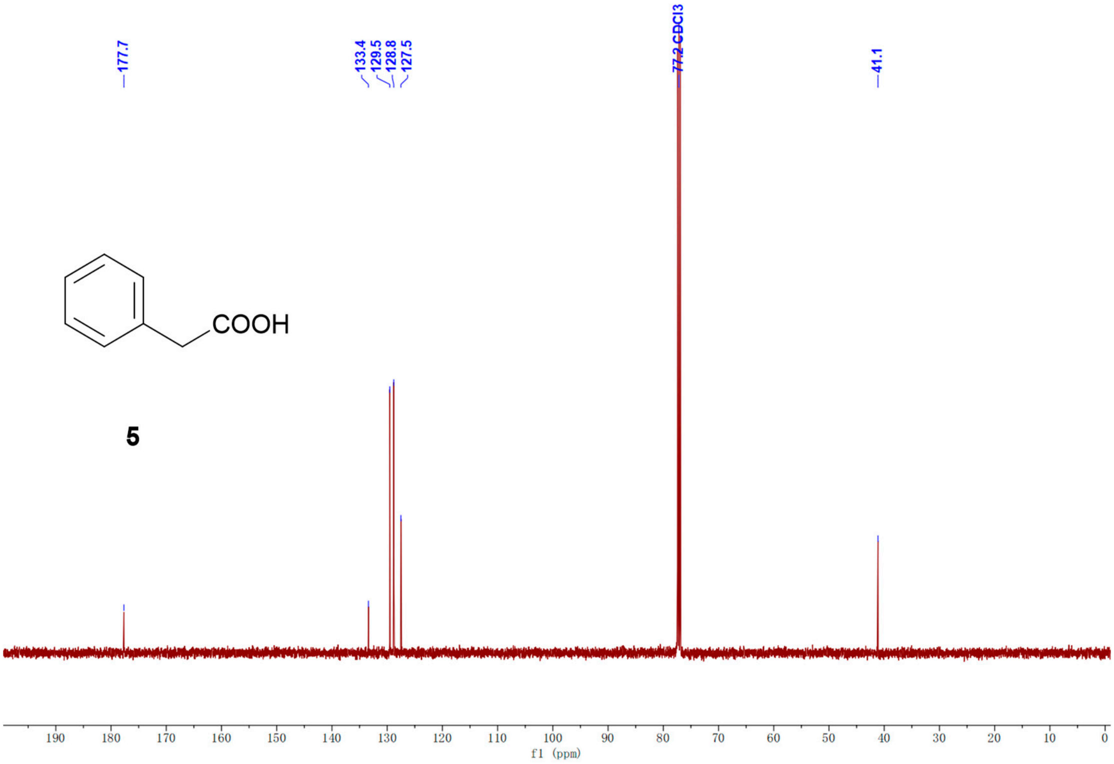

Figure S10 ^{13}C NMR spectra of compound **5** (Recorded at 125 MHz in $CDCl_3$)

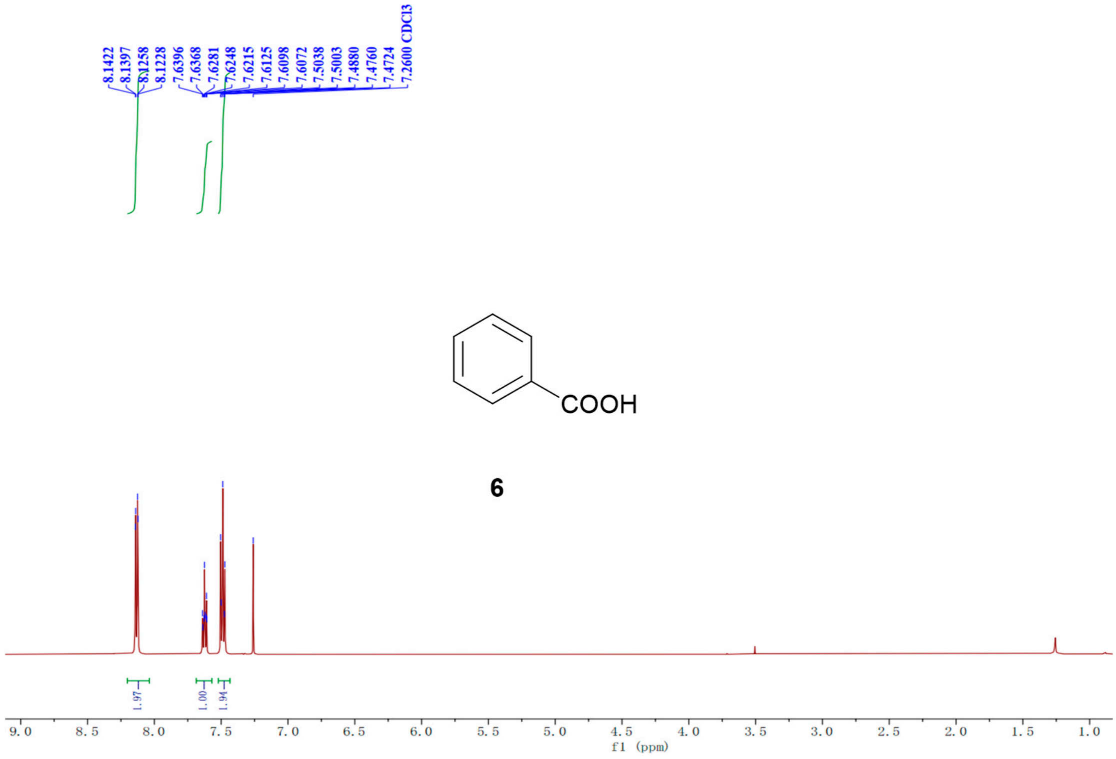

Figure S11 ^{1}H NMR spectra of compound **6** (Recorded at 500 MHz in $CDCl_3$)

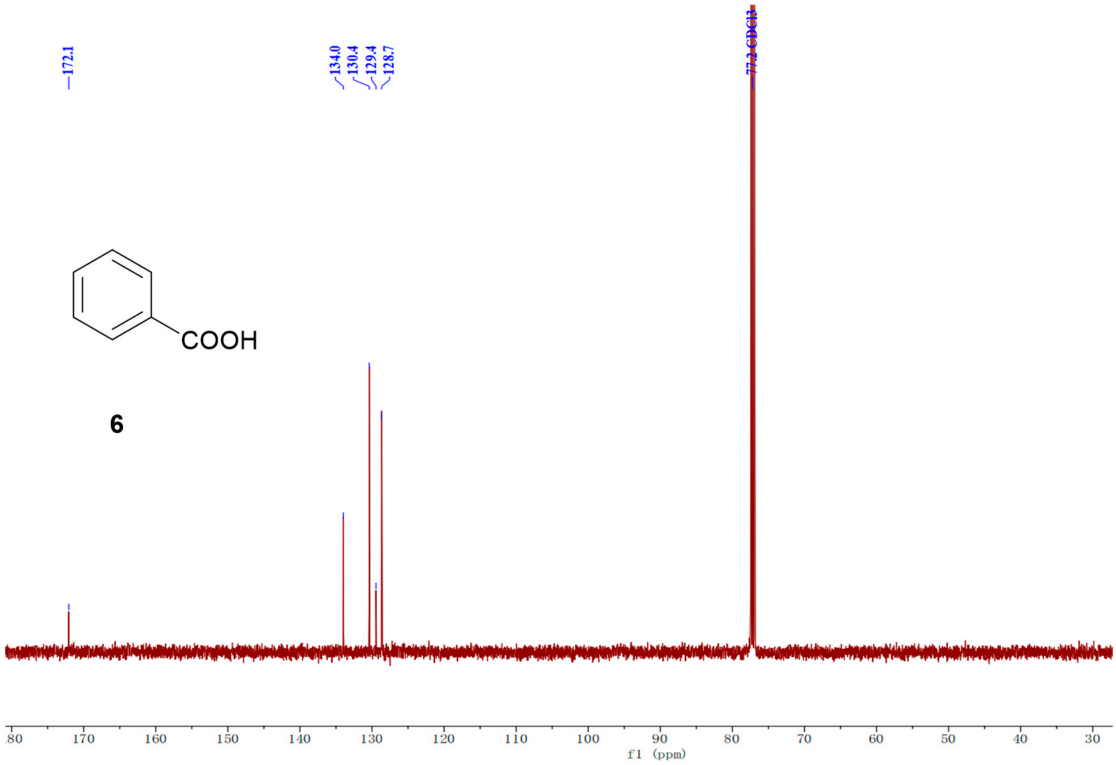

Figure S12 ^{13}C NMR spectra of compound **6** (Recorded at 125 MHz in $CDCl_3$)

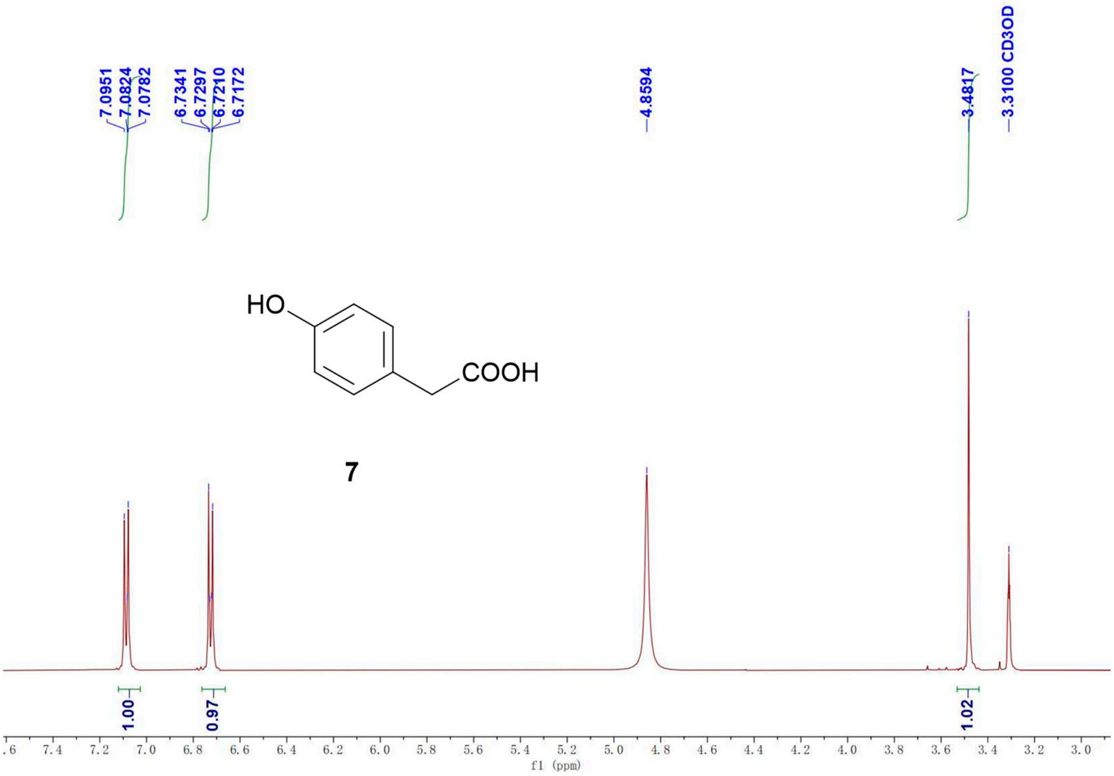

Figure S13 ^{1}H NMR spectra of compound **7** (Recorded at 500 MHz in CD_3OD)

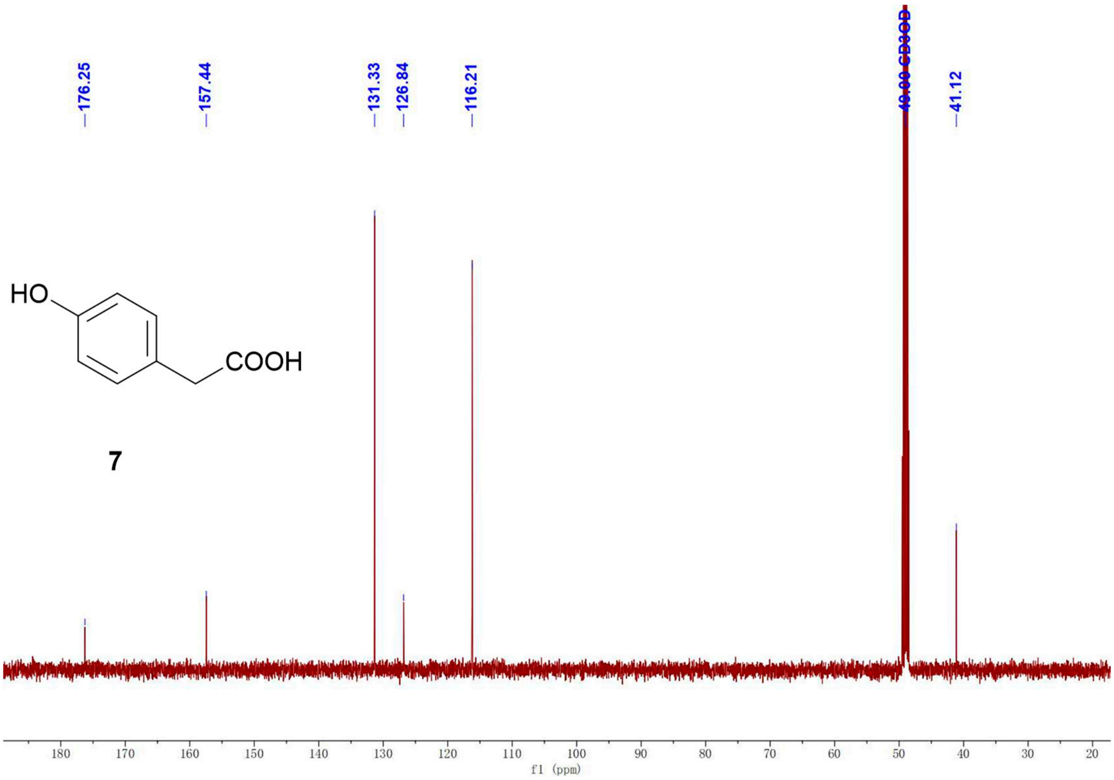

Figure S14 ^{13}C NMR spectra of compound **7** (Recorded at 125 MHz in CD_3OD)

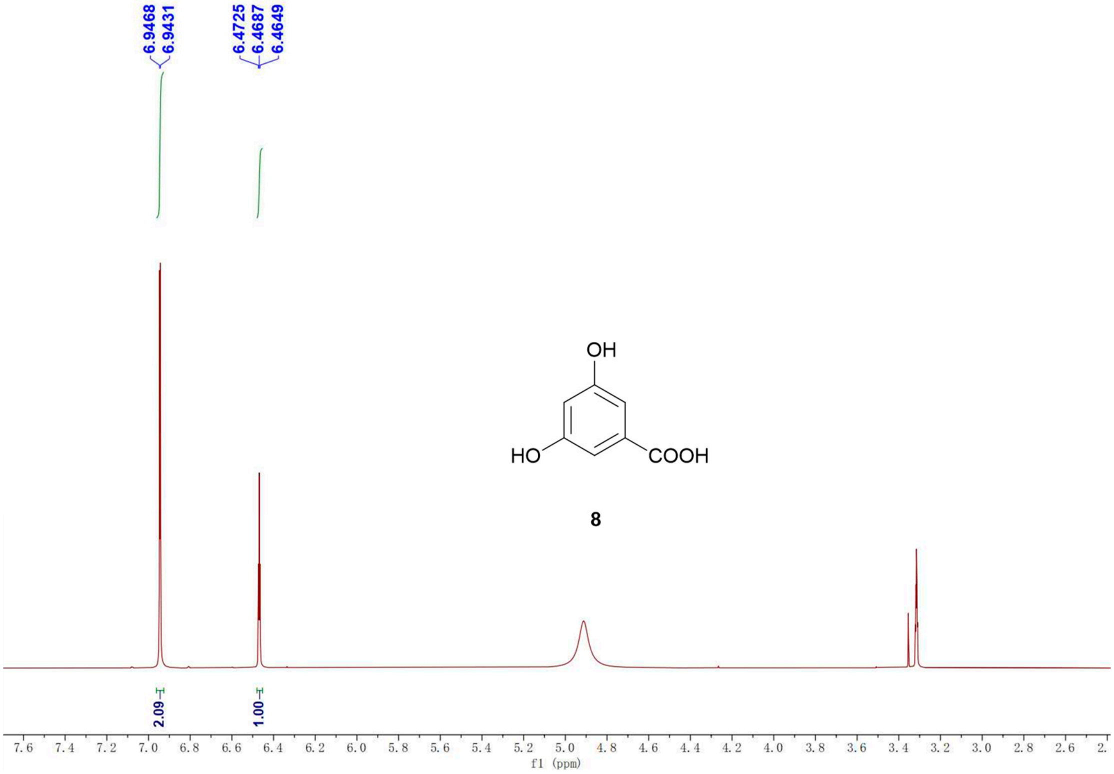

Figure S15 ^{1}H NMR spectra of compound **8** (Recorded at 600 MHz in CD_3OD)

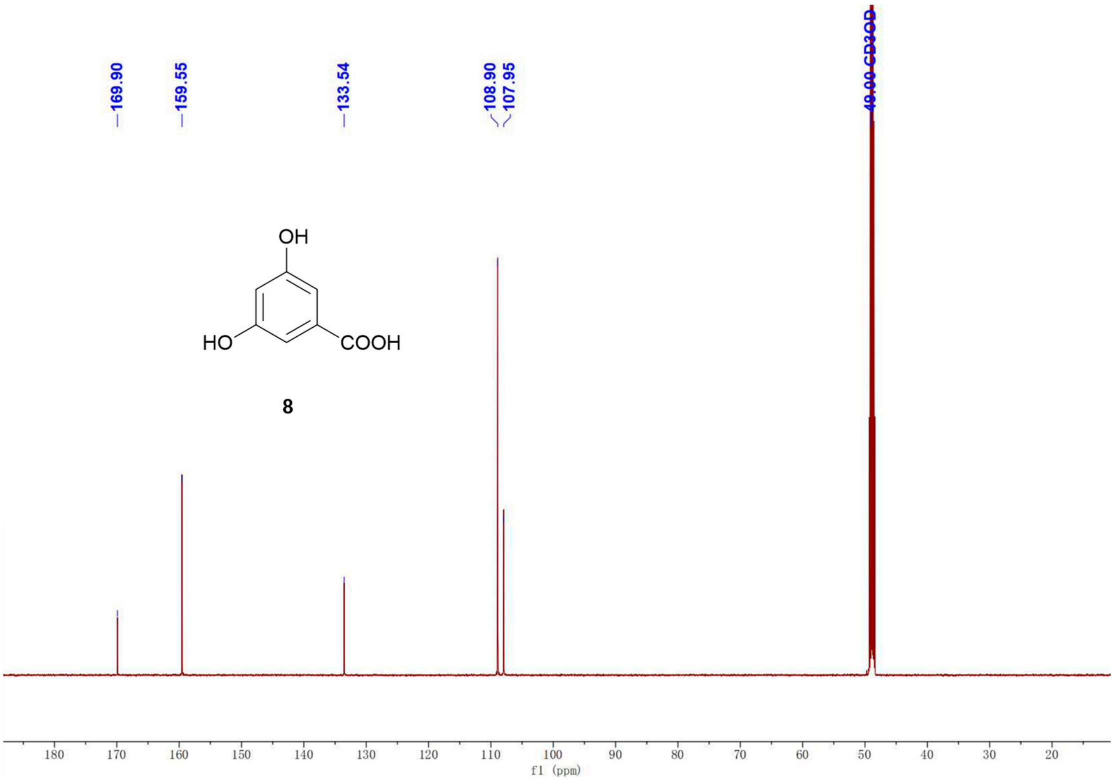

Figure S16 ^{13}C NMR spectra of compound **8** (Recorded at 150 MHz in CD_3OD)

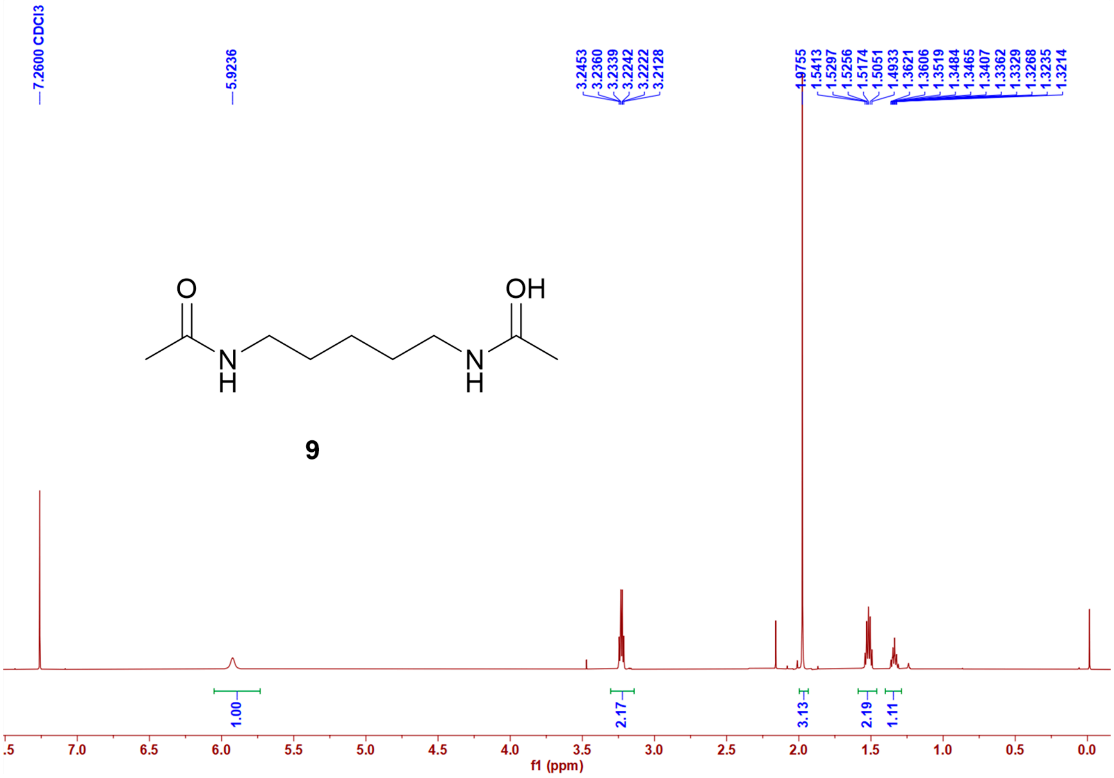

Figure S17 ¹H NMR spectra of compound **9** (Recorded at 600 MHz in $CDCl_3$)

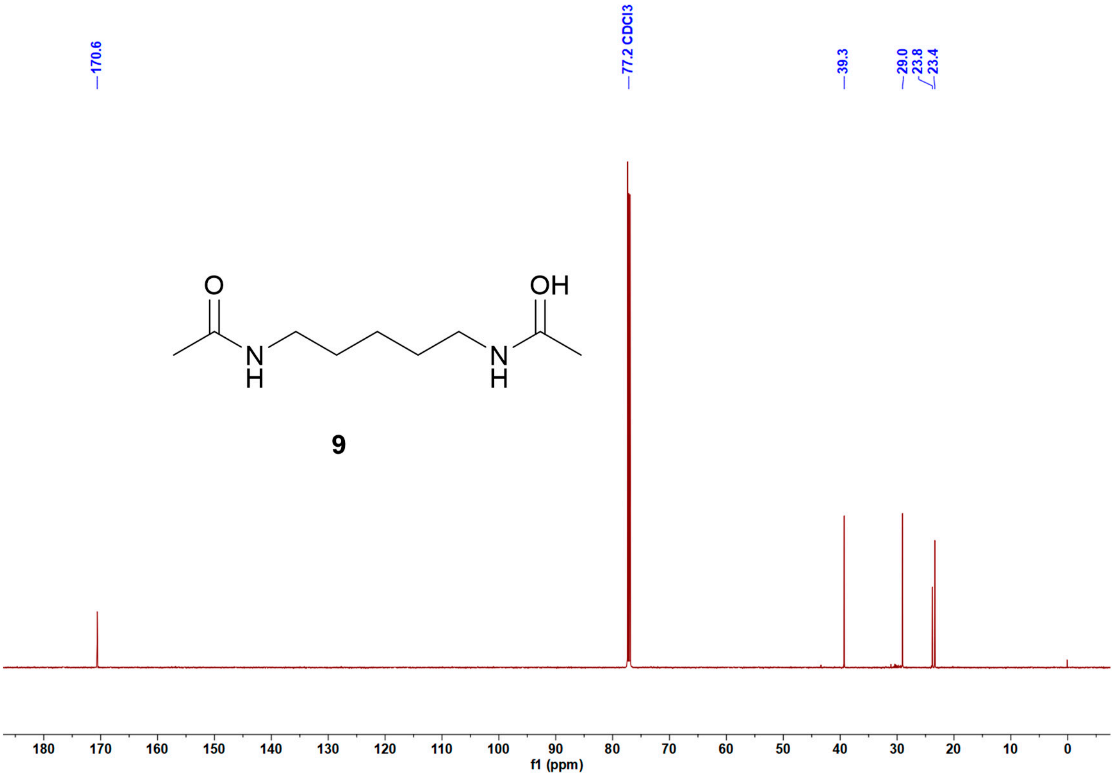

Figure S18 ^{13}C NMR spectra of compound **9** (Recorded at 150 MHz in $CDCl_3$)

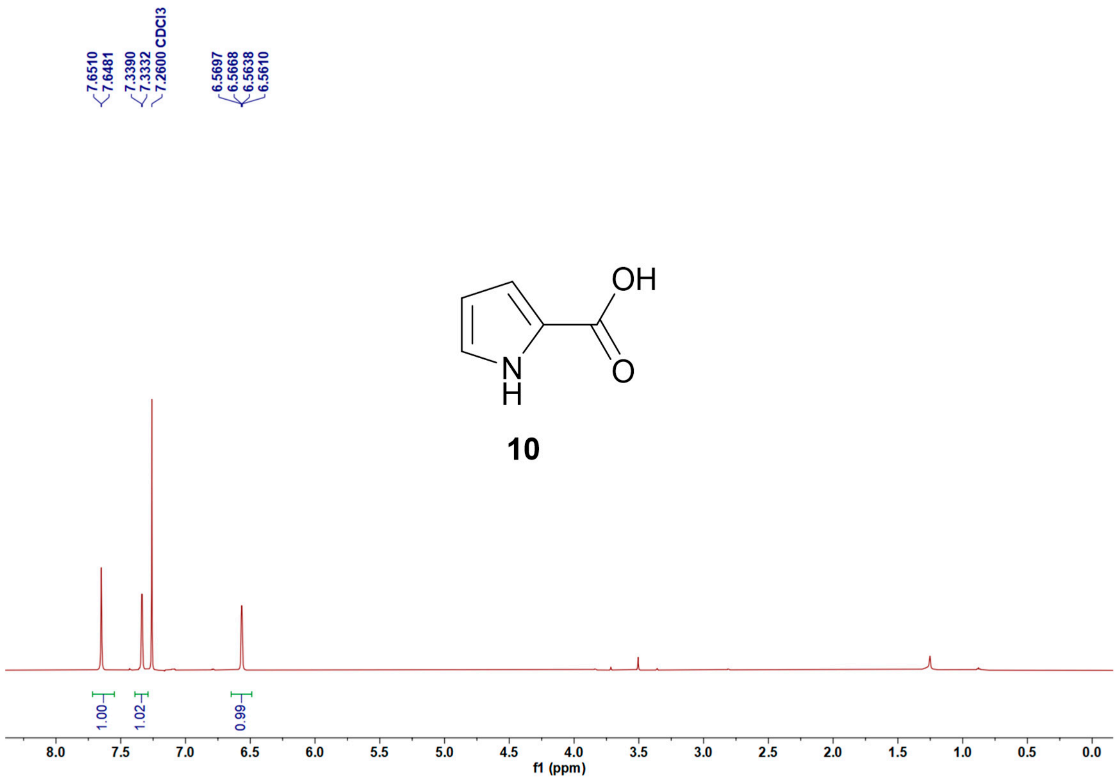

Figure S19 ^{1}H NMR spectra of compound **10** (Recorded at 600 MHz in $CDCl_3$)

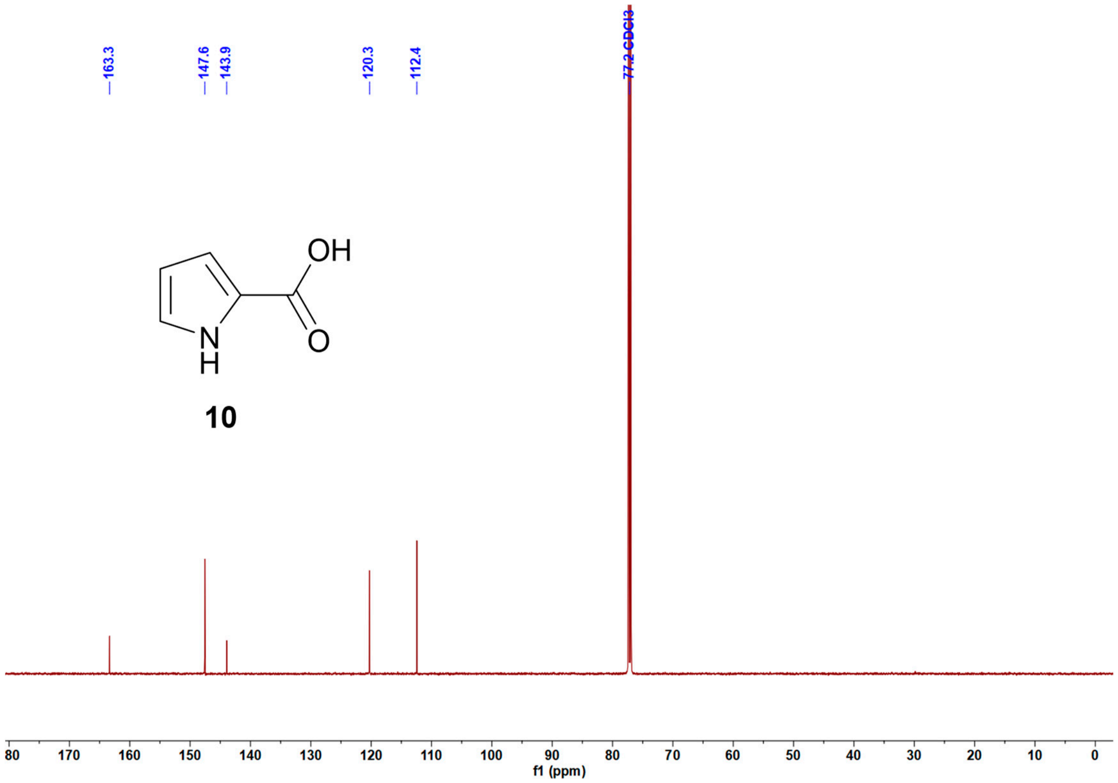

Figure S20 ^{13}C NMR spectra of compound **10** (Recorded at 150 MHz in $CDCl_3$)

TTCACTCGCCGTTACTGAGGCAATCCCTGTTGGTTTCTTTTCCTCCGCTTATTGATATGCTTAAGTT
CAGCGGGTATCCCTACCTGATCCGAGGTCAAATTGTGTATGGTGGATGCTAGGCCACGAGGGTCC
TGAAAAAAGGGCTCCAATTTACGCCCGCAACTGCGACCGGTGTGCCATCGGCGTATGGAGAAA
GGTGAGCATTTTACTGCAAGCCTATATATCCCATAACTCCACGGTTGATGGCCTCGGTGCTGTCGC
ATCTGGAGGCGGGTCTACGTCTATTTAGGACATTGGGAAACTAATTGCCAATCGCTATCCCATTAG
GCCAAAACCCCCCAACGATAGTAATCAAACCCGATGGGGGAGGAGGTTTTTATGACGCTCGAAC
AGGCATGCCCCCCGGAATACCAGGGGGCGCAATGTGCGTTCAAAGATTCGATGATTCACTGAAT
TCTGCAATTCACATTACTTATCGCATTTCGCTGCGTTCTTCATCGATGTGGGAACCAAGAGATCCG
TTGTTGAAAGTTTTAACTTTTTTGTTTTGTTATGATTCTGACGTCGGCTTGTTCACAAAGAGTTTTG
GTTGTTGTTCCTCCCCCCAGCGGGTAGCCGGGGGAAGCAAGGCGGGACAGGTACGCAGAGGGT
TTAGATGGGGGCGGCTCCGGGTCCGGCCAGGACGTTCAACAACGTAAAGCTACTAGCCCTGGTG
GCCCCTCGGCTGCCCTTTTCTGTGTGGTTCTTGGTAATGATCCTTCCGCAGGTTCACCTACG

Figure S21 ITS DNA sequence of the strain *Morchella importuna*
